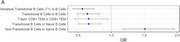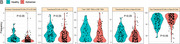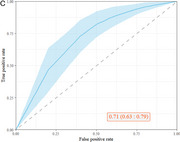# Discriminative features for Alzheimer disease based on peripheral blood deep‐immunophenotyping

**DOI:** 10.1002/alz70855_102619

**Published:** 2025-12-23

**Authors:** Yang Feng, Rafael Veiga, Lidia Yshii, Julika Neumann, Teresa Prezzemolo, Emanuela Pasciuto, Stephanie Humblet‐Baron, Adrian Liston, Rik Vandenberghe

**Affiliations:** ^1^ Laboratory for Cognitive Neurology, KU Leuven, Leuven, Belgium; ^2^ Department of Pathology, University of Cambridge, Cambridge, United Kingdom; ^3^ Laboratory for Neuroinflammation, Department of Neurosciences, KU Leuven, Leuven, Belgium; ^4^ Department of Microbiology, Immunology and Transplantation, KU Leuven, Leuven, Belgium; ^5^ Flanders Institute for Biotechnology (VIB), Antwerp, Belgium; ^6^ University of Antwerp, Antwerp, Belgium; ^7^ Department of Pathology, University of Cambridge, Cambridge, United Kingdom; ^8^ University Hospitals Leuven, Leuven, Belgium; ^9^ Laboratory for Cognitive Neurology, KU Leuven, Leuven, Leuven, Belgium; ^10^ Alzheimer Research Centre KU Leuven, Leuven Brain Institute, Leuven, Belgium

## Abstract

**Background:**

Immunity is a key driving factor in pathogenesis and progression of Alzheimer's disease. Here we examine the peripheral changes in both innate and adaptive immunity in AD and healthy subjects between 50 and 80 years old by immunophenotyping and systems immunology.

**Method:**

184 AD patients (mean age 69.4 ± 6.9 years; 88 males and 96 females; MMSE range 10–30) and 105 healthy spouses (mean age 65.3 ± 7.9 years; 50 males and 55 females) belong to a memory clinic‐recruited cohort. PBMC were collected and applied to high‐dimensional flow cytometry and auto‐gated pipeline, covering a comprehensive innate and adaptive immune cells. Data were processed by data cleaning procedure, standardization and Box‐Cox transformation. Logistic regression analysis was performed with adjusting age/gender and excluding the interaction of age. Mann‐Whitney *U* test was performed to compare differences between two groups, with Benjamini‐Hochberg correction applied to control the false discovery rate. A binary classification machine learning model was developed using logistic regression with L2 regularization. Average AUC value was calculated by repeating 10‐fold cross‐validation 100 times.

**Result:**

Immature Transitional B Cells (T1) in B Cells (OR=0.56,95% CI: 0.42‐0.74, *p* <0.001), Transitional B Cells in B Cells (OR=0.63,95% CI: 0.49‐0.82, *p* <0.001), and Transitional B Cells in Naive B Cells (OR=0.66,95% CI:0.51‐0.85, *p* = 0.001), are negatively associated with the risk of AD. (Figure 1A). Compared with healthy controls, Immature Transitional B Cells (T1) in B Cells, Transitional B Cells in B Cells and Transitional B Cells in Naive B Cell (*p* <0.001, FDR<0.05) are all significantly reduced in AD patients (Figure 1B) while Non‐Transitional B Cells in Naive B Cells significantly increases (*p* = 0.001, FDR<0.05). The machine learning model shows a good discriminative ability of AD with the average AUC value of 0.71 (95% CI:0.63‐0.79) using the optimal number of top four features (Figure 1C).

**Conclusion:**

Abnormalities in peripheral adaptive immunity in AD principally occur in the premature B cell compartment.